# Molecular crosstalk between gas transmitters and catecholamine biosynthesis pathways: a narrative review

**DOI:** 10.4103/mgr.MEDGASRES-D-26-00004

**Published:** 2026-09-12

**Authors:** Robert Dingley, Sujeenthar Tharmalingam, T. C. Tai

**Affiliations:** 1 Medical Sciences Division, Northern Ontario School of Medicine University, Sudbury, ON, Canada; 2 School of Natural Sciences, Laurentian University, Sudbury, ON, Canada; 3 Health Sciences North Research Institute, Sudbury, ON, Canada

**Keywords:** adrenal, carbon monoxide, catecholamines, dopamine, endocrine, epinephrine, gas transmitter, hydrogen sulfide, nitric oxide, norepinephrine

## Abstract

Catecholamines, particularly the hormone and neurotransmitter epinephrine, are important regulators of blood pressure and cardiovascular function. The expression of genes encoding catecholamine biosynthetic enzymes is subject to strict transcriptional regulation through both hormonal and neuronal mechanisms. These genes are also regulated by hypoxia and reactive oxygen species. A class of small gaseous transmitters, known as gas transmitters, serve as potent regulators of blood pressure and hypoxic signaling; however, their roles in catecholamine biosynthesis remain poorly understood. This manuscript reviews the current state of catecholamine and gas transmitter research, particularly their interactions in relation to hypertension. Current evidence shows that the gas transmitters nitric oxide, carbon monoxide, and hydrogen sulfide modulate catecholamine biosynthesis and release through both shared and distinct mechanisms. This regulation is highly dependent on concentration, cellular context, and mode of delivery. Additionally, this review explores hypoxic signaling as a link between catecholamines and gas transmitters. Together, these findings highlight a complex and context-dependent regulatory signaling between gas transmitters and catecholamines. Further mechanistic studies investigating endogenous signaling are required to elucidate these interactions and their therapeutic potential.

## Introduction

Despite being a highly prevalent disease, the etiology of essential hypertension is not fully understood.^1^ There is likely no single common cause of essential hypertension; instead, it should be viewed as a multifactorial disease caused by the interaction of multiple environmental and genetic factors.^1^ Therefore, it is more likely that understanding the etiology of essential hypertension depends on many genes involved in each determinant of blood pressure.^2^ Genome-wide association studies have identified over 1000 common single-nucleotide polymorphisms associated with blood pressure regulation.^3^ Therefore, understanding the etiology of hypertension requires investigating all the factors that regulate blood pressure.

One key feature of the factors that regulate blood pressure is their interdependence.^1^ To that end, a detailed investigation of these factors and their interactions is key to understanding hypertension. Among the key factors regulating blood pressure are catecholamines and gas transmitters, also called gasotransmitters, two classes of signaling molecules that have traditionally been studied independently. Catecholamines are a class of organic molecules consisting of a catechol (1,2-dihydroxybenzene) group and a side-chain amino acid. When released into circulation, they can act as hormones or as neurotransmitters when released directly by neurons.^4^ Catecholamines are also central mediators of the cardiovascular system by modulating cardiac output and vascular tone through autonomic signaling pathways.^4^,^5^,^6^ Dysregulation of catecholamine signaling contributes to pathological development of hypertension and other cardiovascular diseases.^5^,^6^

Gas transmitters are a classification of gases that act as neurotransmitters and have emerged as key regulators of cellular signaling and vascular physiology.^7^ Currently, nitric oxide (NO), carbon monoxide (CO) and hydrogen sulfide (H_2_S) are considered gas transmitters.^7^ These small diffusible molecules function as signaling mediators, regulating cellular redox state, mitochondrial function, and metabolic activity.^7^,^8^ Growing evidence suggests that these gases form an interconnected signaling network that regulates vascular tone and cardiovascular health.^7^,^8^,^9^

While both the effects of catecholamines and gas transmitters on blood pressure and hypertension have been studied, the crosstalk between these molecules and their relevance to blood pressure regulation is less understood. This review aimed to explore the regulation of catecholamine biosynthesis and the roles of the gas transmitters NO, CO, and H_2_S, especially where these two systems intersect. Additionally, this review also highlighted hypoxic signaling as a potential link between hypertension, catecholamines, and gas transmitters.

## Search Strategy

Relevant literature was retrieved through electronic searches of the PubMed database from August 2025 to May 2026 with the following search terms (including abbreviations): gas transmitters, gasotransmitters, carbon monoxide, hydrogen sulfide, nitric oxide, catecholamines, epinephrine, norepinephrine, dopamine, phenylethanolamine N-methyltransferase, tyrosine hydroxylase, and dopamine beta-hydroxylase. Articles that were not available in English were excluded. Relevance was determined by the content in the title and abstract. If relevant, the entire paper was then accessed to ensure that interactions between catecholamines and gas transmitters were discussed. Given the limited number of recent studies directly examining catecholamine–gas transmitter interactions, this review integrates foundational experimental work with more recent findings to provide a current overview of the field.

## Catecholamines

Catecholamine biosynthesis primarily takes place in the adrenal medullary chromaffin cells and post-ganglionic fibers of the sympathetic nervous system. However, biosynthetic enzymes for catecholamines are also localized in various other tissues and organs, such as the central nervous system, heart,^10^,^11^ lung,^12^ and spleen.^13^ The catecholamines dopamine and norepinephrine play a major role in the central nervous system as neuromodulators.^14^ The catecholamines norepinephrine and epinephrine are vital to the physiological response to stressors, in the propagation of the “fight or flight” response.^15^

Catecholamine response to stressors is driven by two major pathways: the hypothalamic–pituitary–adrenal axis, which is stimulated through a hormonal mechanism, and the sympathoadrenal (SA) system, which is stimulated through a neural mechanism.^16^ The hypothalamic–pituitary–adrenal axis is a major neuroendocrine system that regulates stress response and controls many other physiological processes, including digestion, immune response, mood, and emotions.^17^ It includes the paraventricular nucleus of the hypothalamus, the anterior pituitary gland, and the adrenal gland and facilitates the activation of catecholamine systems through hormonal mechanisms.^17^ Hypothalamic–pituitary–adrenal axis activation induces a rapid increase in the biosynthesis and secretion of glucocorticoids in the adrenal cortex, mainly cortisol, in humans. The adrenal medulla is exposed to very high concentrations of glucocorticoids.^18^ Glucocorticoids directly increase the release of catecholamines from the adrenal medulla^19^ and increase both enzyme activity and expression of catecholamine biosynthesis genes.^20^ The SA system consists of direct innervation of the adrenal medulla by the sympathetic nervous system.^17^ The effects of the SA are quicker than those of the hypothalamic–pituitary–adrenal axis, which has longer-lasting but slower effects.^21^ Stress signals are sent to the preganglionic sympathetic neurons, which then project to the adrenal cortex and medulla via the splanchnic nerves.^17^ The splanchnic nerves release acetylcholine alongside several other neurotransmitters and neuropeptides, such as pituitary adenylate cyclase-activating peptide, neuropeptide Y and vasoactive intestinal peptide.^16^ Acetylcholine induces catecholamine release from chromaffin cells by acting on nicotinic and muscarinic receptors, enhancing Ca^2+^ entry and supporting exocytosis.^22^ Neuropeptide Y and vasoactive intestinal peptide, released alongside acetylcholine comodulate the significant increase in catecholamine release through Ca^2+^-mediated action potential.^16^ Acetylcholine and pituitary adenylate cyclase-activating peptide are also integral to the induction of catecholamine biosynthesis.^23^

The catecholamines dopamine, norepinephrine and epinephrine (also known as noradrenaline and adrenaline) are all produced in the same sequential enzymatic pathway^24^ as shown in **Figure 1**. The process of catecholamine biosynthesis initiates with the essential amino acid L-tyrosine or by the conversion of L-phenylalanine to L-tyrosine. This conversion is achieved by the addition of a hydroxyl group, catalyzed by the aromatic amino acid hydroxylase enzyme phenylalanine 4-hydroxylase. Subsequently, L-tyrosine is converted into L-3,4-dihydroxyphenylalanine (L-DOPA) by the addition of another hydroxyl group catalyzed by a separate aromatic amino acid hydroxylase enzyme, tyrosine 3-hydroxylase (TH). TH is traditionally considered the rate-limiting enzyme of catecholamine biosynthesis.^25^ L-DOPA is then converted to dopamine by removal of the carboxyl group, catalyzed by aromatic L-amino acid decarboxylase (AADC).^26^ Following this, dopamine is converted to norepinephrine by the addition of a hydroxyl group catalyzed by the enzyme dopamine β-hydroxylase (DBH).^26^ Finally, norepinephrine is converted into epinephrine by adding a methyl group onto the amine group, catalyzed by the enzyme phenylethanolamine N-methyltransferase (PNMT).^26^ The sequential nature of the catecholamine biosynthetic pathways means that each enzyme is subject to independent regulation, and therefore, each product can be individually regulated.^26^ The key transcription factors involved in the transcriptional regulation of catecholamine biosynthesis are shown in **Figure 2**.

**Figure 1:**
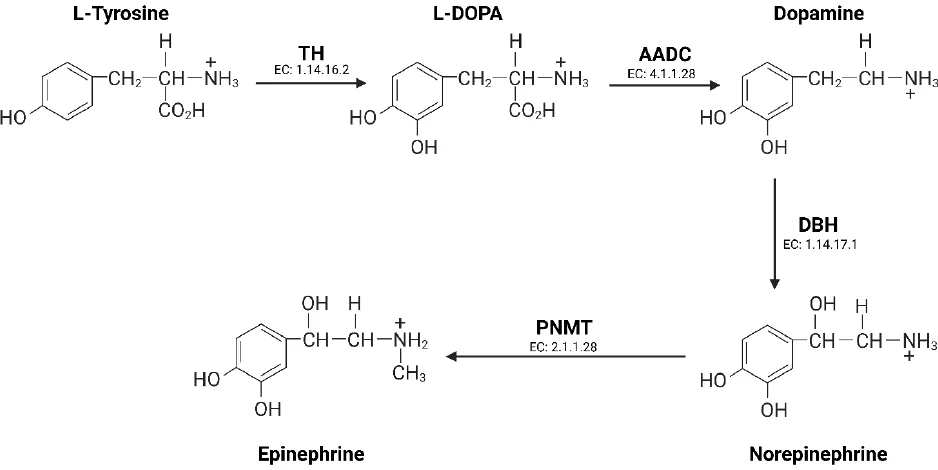
Catecholamine biosynthesis pathway. The catecholamines dopamine, norepinephrine, and epinephrine are synthesized in a sequential enzymatic biosynthetic pathway beginning with L-tyrosine. Created in BioRender. Dingley R. (https://BioRender.com/sbd1rgt) is licensed under CC BY 4.0. AADC: Aromatic L-amino acid decarboxylase; DBH: dopamine β-hydroxylase; L-DOPA: L-3,4-dihydroxyphenylalanine; PNMT: phenylethanolamine N-methyltransferase; TH: tyrosine 3-hydroxylase.

**Figure 2:**
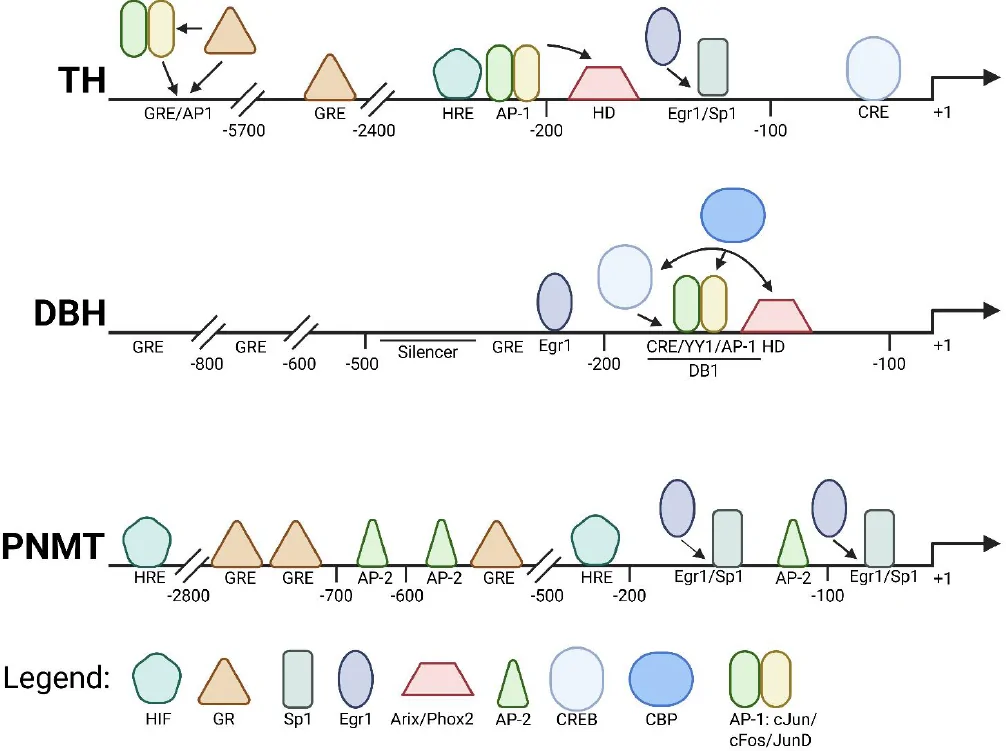
Significant motifs and related transcription factors of the *Th*, *Dbh*, and *Pnmt* promoters. The approximate positions of some of the various regulatory motifs and some of the transcription factors observed interacting with these sites are shown. Created in BioRender. Dingley R (https://BioRender.com/dc5otav) is licensed under CC BY 4.0. AP: Activator protein; CBP: CREB binding protein; CRE: cyclic adenosine monophosphate response element; CREB: CRE binding protein; Dbh: dopamine β-hydroxylase; Egr1: early growth response factor 1; GR: glucocorticoid receptor; GRE: glucocorticoid response element; HIF: hypoxia-inducible factor; HRE: hypoxia response element; Pnmt: phenylethanolamine N-methyltransferase; Sp1: specificity protein 1; Th: tyrosine 3-hydroxylase.

### Tyrosine 3-hydroxylase

TH is the first enzyme in the classical catecholamine biosynthesis pathway and acts as the major rate-limiting step in the pathway.^27^ TH catalyzes the conversion of L-tyrosine to L-DOPA using oxygen (O_2_), Fe^2+^, and tetrahydrobiopterin.^27^ The rate of TH is typically limited by the availability of tetrahydrobiopterin, which is present at sub-saturating levels and is converted into tetrahydrobiopterin-4a-carbinolamine by TH and, therefore, must be recycled back before re-involvement in more production of L-DOPA.^28^ Short-term TH activity is regulated mainly by feedback inhibition by catecholamines and phosphorylation.^16^ TH is a homotetramer with the four subunits coordinating with an iron (II) atom at the active site. Oxidation to iron (III) inactivates the enzyme.^29^ Dopamine, norepinephrine, and epinephrine bind to the active site of TH only when its iron is oxidized, effectively trapping the enzyme in its inactive state, acting as a form of feedback inhibition in catecholamine biosynthesis.^30^ The binding of dopamine specifically has been identified to prohibit the entry of substrates into the active site.^25^ TH is also regulated by the phosphorylation of multiple serine (Ser) residues in its regulatory domain: Ser8, Ser19, Ser31, and Ser40.^31^ The phosphorylation of all the Ser residues except Ser8 is increased in conditions, producing more catecholamines.^30^ Ser31 and Ser40 phosphorylation increase TH activity, while Ser8 and Ser19 phosphorylation do not directly affect TH activity.^32^ Ser40 phosphorylation, primarily by protein kinase A (PKA), results in a 300-fold decrease in affinity towards dopamine.^30^ Therefore, Ser40 phosphorylation represents a mechanism to ameliorate the feedback inhibition of catecholamine biosynthesis on TH, by increasing its affinity to BH4 and reducing the iron back to its ferrous form, activating TH.^30^ While the kinases and phosphatases involved in TH phosphorylation/dephosphorylation have been well studied,^32^ the cellular signaling cascades responsible need to be more defined. Conflicting results on the involvement of intracellular Ca^2+^ levels in TH have been shown.^33^,^34^ A novel H^+^ signaling method has also been proposed.^35^

TH activity is also subject to transcriptional regulation. The *Th* promoter contains several regulator domains known to several regulatory proteins, including hypoxia-inducible factor (HIF), activating protein-1 (AP-1) and -2 (AP-2), early growth factor (Egr1), specificity protein 1 (Sp1) and the cyclic adenosine monophosphate response element (CRE) binding proteins (CREB).^36^,^37^,^38^ This CRE is important in the transcription of basal and cAMP-induced *Th*.^39^ AP-1 factors, including c-Fos, have been implicated in hormonal, neuronal, and hypoxia-mediated regulation of *Th*.^40^,^41^ The *Th* promoter also contains homeodomain core recognition (HD) sites proximal to the AP-1 motif.^42^,^43^ These HD sites serve as binding sites for Arix/Phox2 proteins.^44^ Arix/Phox2 proteins have been implicated in nervous system development, particularly in noradrenergic neuron differentiation.^45^ Another AP-1-like motif was found at –5734 to –5728 bp in the promoter of rat *Th*, identified as a glucocorticoid response element (GRE) despite having little similarity to known GREs.^46^ A more canonical GRE was initially identified at –454 bp in the *Th* promoter, but appears to be non-functional.^37^ GREs are acted on directly by glucocorticoid receptors (GRs), which bind directly to canonical GREs when bound to their ligand, activating transcription.^47^ GRs can also bind to other transcription factors, such as AP-1, known as tethering, and serve as a potent repressor of these factors.^47^ The GRE/AP-1 sequence in the *Th* promoter showed binding of both AP-1 and GRE-binding protein.^46^ This suggests that the GRE/AP-1 sequence may regulate *Th* expression by direct GR binding despite having little homology with the typical GRE.^48^ Additionally, another GRE motif, –2435 to –2421 bp, has been implicated in the canonical glucocorticoid-mediated induction of *Th*.^49^ The *Th* promoter contains three different hypoxia response elements (HREs); however, only one is necessary for the transcriptional response to hypoxia.^50^,^51^ HREs are bound by HIFs, which are stabilized and localized to the nucleus without oxygen; both HIF1α and HIF2α affect the *Th* promoter.^51^ The AP-1 and CRE motifs also regulate *Th* transcription in response to hypoxia.^40^,^50^ AP-1 protein c-Fos expression is increased in response to intermittent hypoxia and can then increase *Th* expression.^52^

### Aromatic-L-amino acid decarboxylase

L-DOPA is converted into dopamine by the decarboxylation catalyzed by the activity of a nonspecific enzyme AADC in the presence of its cofactor pyridoxal phosphate.^16^ AADC is an essential enzyme for synthesizing catecholamines, indoleamines, and trace amines. It is not the limiting factor in the catecholamine biosynthesis pathway.^53^ Despite its general ubiquity, AADC is still subjected to post- and pre-translation regulation.^53^ PKA and cyclic guanosine monophosphate (cGMP)-dependent protein kinase G can phosphorylate AADC, significantly increasing its enzymatic activity.^54^,^55^ AADC is regulated on the gene level by alternative promoters on a tissue-specific basis.^56^ AADC promoter contains two putative HREs, and regulation via those HREs appears vital to embryonic development of catecholaminergic systems.^57^

### Dopamine β-hydroxylase

Dopamine is converted into norepinephrine by hydroxylation catalyzed by DBH in the presence of vitamin C (ascorbate/ascorbic acid) and O_2_. DBH is the only catecholamine biosynthesis enzyme found in membrane-bound forms. DBH can also exist in soluble form, and the levels of expression and activity are similar for both the soluble and membrane-bound forms of DBH.^58^ Once synthesized in the endoplasmic reticulum, DBH is packaged into granulated vesicles, where it synthesizes norepinephrine.^59^ Soluble bovine DBH activity is high in these secretory vesicles due to the internal pH (5.5–5.7), maintaining the enzyme’s dimeric form.^60^ Soluble bovine DBH is released into the bloodstream during vesicle exocytosis, where it is most stable in a tetrameric form with less affinity toward dopamine.^60^,^61^ Interestingly, this pH-dependent conversion is not observed in soluble human DBH.^62^ DBH is a copper monooxygenase whose activity depends on the availability of both ascorbate and copper (Cu^2+^) ions.^63^,^64^ Intravascular ascorbate levels are maintained by recycling by electron transport across the vesicle membrane from cytosolic ascorbate.^64^ While the exact mechanism of DBH enzyme activity has not been studied in detail, it is proposed to be very similar to the mechanisms of the homologous peptidylglycine α-hydroxylating monooxygenase enzyme.^65^ The catalytic activity of both DBH and peptidylglycine α-hydroxylating monooxygenase appeared very similar,^66^ and human DBH crystallography has confirmed these two enzymes’ similarities.^62^

*Dbh* is subject to tight transcriptional control, and the enhancers and inhibitors located on the promoter of the *Dbh* gene are well documented. The *Dbh* promoter contains a multifunctional region (DB1) that regulates basal, phorbol ester, and cAMP-mediated expression.^67^ A CRE was identified in the DB1 region; overlapping this CRE are AP-1 and *Yin*-*Yang* 1 (YY1) motifs.^68^ The YY1 element positively regulated basal expression of *Dbh* while decreasing cAMP-mediated induction of *Dbh* expression, possibly due to competitive binding between YY1 and other factors.^68^,^69^ The induction of *Dbh* expression by cAMP is coordinated by AP-1 proteins instead of CREB alone.^42^ After cAMP treatment, the factors bound to the CRE/YY1/AP-1 element switch from c-Jun and JunD to c-Fos, c-Jun, and JunD.^42^ Moreover, c-Fos itself is regulated by cAMP through phosphorylation by PKA.^70^ The DB1 region also contains two HD sites, HD1 and HD2, and a third HD proximal to DB1 (HD3).^44^ Arix exhibits synergistic interaction with cAMP-mediated induction of *Dbh* expression.^71^ Arix is a critical tissue-specific regulator of *Dbh* expression, and the interaction between Arix proteins bound to the HDs located in and proximal to DB1 is required for the maximal response to PKA. Furthermore, the interaction between Arix and CREB-binding protein and AP-1 proteins is required for this maximal response.^44^ This AP-1-dependent Arix binding is a key similarity between the *Dbh* and *Th* promoters. Several putative GREs have been identified in the *Dbh* promoter, and *Dbh* expression does increase in response to glucocorticoids.^72^ However, the functionality of any specific GRE has yet to be proven. An Egr1 motif has been identified in the *Dbh* promoter, and interestingly, it does not induce but inhibits *Dbh* expression.^73^ The *Dbh* promoter includes a cell-specific silencer region, with multiple putative binding sites for negative regulatory elements.^74^ While the expression of *Dbh* is induced by intermittent hypoxia,^75^,^76^ no putative HREs have been found.

### Phenylethanolamine N-methyltransferase

PNMT is the terminal enzyme in the catecholamine biosynthesis pathway. PNMT catalyzes the conversion of norepinephrine to epinephrine via the transfer of the methyl group from S-adenosyl-L-methionine (SAM) to norepinephrine.^27^ In the absence of a substrate, the binding of SAM also serves as a critical stabilizing agent for PNMT.^77^

The production and activity of PNMT are highly dependent on glucocorticoids.^78^ The regulation of PNMT by glucocorticoids occurs through several mechanisms, including increases in PNMT enzyme activity,^20^ stabilization of PNMT by elevating the concentration of SAM,^78^,^79^ augmentation of the amount of the active intronless mRNA splice variant of PNMT,^20^ and promotion of *Pnmt* gene transcription.^19^,^80^ Three functional GREs have been identified in the promoter region of rat Pnmt.^81^ The rat *Pnmt* promoter contains two Egr1 sites.^82^ With one of those two sites being implicated in the *Pnmt* promoter responsiveness to cAMP-dependent protein kinase-mediated transcription, as no canonical CREs have been identified.^83^ The Egr1 sites also overlap with Sp1 sites.^84^ Sp1 has also been implicated in the post-translational modification of *Pnmt* mRNA, promoting the active intronless variant.^85^ The rat *Pnmt* promoter contains three AP-2 sites, which mediate promoter activity in a glucocorticoid-dependent manner.^86^ In the rat *Pnmt* promoter, GR, Egr1, and AP-2 activity are synergistic, activating promoter activity beyond the sum of their roles.^87^ Two of the GREs act synergistically with Egr1 and AP-2, and deletion of GREs does not abolish glucocorticoid-induced *Pnmt* expression.^81^,^87^ Dimerization-defective GR mutants also induced *Pnmt* promoter activity, suggesting that the canonical dimerization of GR and subsequent DNA binding is not required to induce *Pnmt* expression.^88^ This secondary mechanism occurs through protein-protein interactions between GR, Egr1 and AP-2 proteins.^81^,^88^ This highlights the *Pnmt* promoter as a combinatorial regulator, integrating hormonal and neuronal signaling to regulate *Pnmt* expression.

Expression of *Pnmt* is also induced by hypoxia,^89^ and the *Pnmt* promoter contains two HREs.^75^ Interestingly, the binding of HIFs to these HREs may not be the primary cause of hypoxia-induced *Pnmt* expression. The overexpression of HIF1α induces the expression of Egr1 and Sp1.^89^ It has been observed that mutation of the Egr1 and Sp1 binding sites completely ameliorates the HIF1α-mediated increase in *Pnmt* promoter activity.^89^ GR expression also increases in PC12 cells in response to hypoxia.^75^ HIF1α induction of GR expression and activity has been demonstrated in another study.^90^ Consequently, it appears probable that HIF1α, Egr1, Sp1, and GR regulate the hypoxia-induced expression of Pnmt. However, in Neuro-2a and NB-1 cells, hypoxia-mediated *Pnmt* expression is driven by miRNA-375-dependent post-transcriptional changes.^76^

## Gas Transmitters

Gas transmitters are unique among neurotransmitters, overturning many conventional criteria of a neurotransmitter.^91^ For instance, as gaseous substances, they are not readily stored in vesicles and, therefore, must be largely synthesized as needed.^91^ Additionally, while the classical neurotransmitter may act through a signaling cascade, these gases can modify intracellular proteins directly.^92^ These differences, among others, highlight the importance of a separate classification scheme for gas transmitters. The criteria for the classification of gas transmitters were first established in 2002 and expanded in 2004, and are as follows: (1) They are small gas molecules; (2) They can freely permeate membranes, meaning they do not depend on membrane receptors; (3) They are produced endogenously and enzymatically, and their production is regulated; (4) They exhibit well-defined specific functions at physiological concentrations, allowing for physiological changes when their endogenous levels are regulated; (5) Their functions can be replicated by exogenous donation; (6) Their cellular effects may or may not be mediated by second messengers, but they should target specific cellular and molecular pathways.^91^,^93^

Gas transmitters have been implicated in a wide array of physical processes, including the regulation of the cardiovascular system, nervous system, immune system, cellular metabolism, gastrointestinal system, and neuroendocrine system, among others.^91^,^94^ The primary mechanism of gas transmitters on these systems is dependent on their chemical reactivity, modifying target activity by post-translational modifications such as S-nitrosylation, sulfhydration, and ligand binding to metalloproteins.^91^ Recently, other gases have been posited to be gas transmitters, such as methane^95^ and cyanide.^96^ Additionally, the term “small molecule signaling agent” has been coined to clarify and widen the criteria to include exogenously received but essential oxygen as a gaseous signaling molecule.^97^ While we acknowledge the widening scope of gas transmitters research, this review will focus on the three established gas transmitters: NO, CO, and H_2_S.

### Nitric oxide

Originally known as “endothelium-derived relaxing factor,” it was then identified as the gas NO, marking the beginning of modern gas transmitter research.^98^,^99^ NO is endogenously synthesized by a family of small multidomain heme-containing proteins called NO synthases (NOSs).^100^ These enzymes catalyze the oxidation of L-arginine into Nφ-hydroxy-L-arginine and then to L-citrulline and NO.^100^ The oxidation requires the cofactors nicotinamide adenine dinucleotide phosphate, flavin adenine dinucleotide, flavin mononucleotide, and tetrahydrobiopterin.^100^ There are several isoforms of NOS, each encoded by a separate gene.^100^ These include the constitutively expressed neuronal NOS, endothelial NOS, and inducible NOS.^100^

NO is unique among the established gas transmitters as it is a free radical, possessing an unpaired electron and does not tend to dimerize under biological conditions.^97^ NO in pure water will generate nitrite (NO_2_^–^) through a readily reversible reaction with nitrogen dioxide (NO_2_) and dinitrogen trioxide (N_2_O_3_) as intermediaries.^97^ Nitrite acts as a stable pool that can be converted back into NO depending on cellular conditions.^101^ N_2_O_3_ can react with thiol groups (R-SH) to form S-nitrosothiols (R-SNO).^97^ S-nitrosylation regulates several biological pathways.^102^ S-nitrosylation typically causes specific physiological changes in protein function; however, the effect on activity varies among target proteins, with both activation and inhibition observed.^103^ S-nitrosylation typically targets specific thiols on a protein to induce a particular effect. For instance, in skeletal muscle calcium channels (RyR1), Cys3635 is the only cysteine among many that, when S-nitrosylated, affects channel activity by allowing for calmodulin-dependent, NO-mediated modulation of channel activity.^104^ Activation of other cysteines results in different changes in channel activity.^105^ S-nitrosylation also affects the activities of several transcription factors, including HIF, Sp1, Sp3, and nuclear factor-κB.^103^ The transcriptional regulatory functions of S-nitrosylation are dose-dependent; physiological levels of NO typically maintain transcription at normal levels, while supraphysiological levels of NO lead to the increased expression of stress response genes and proteins.^103^ For example, HIF1α is stabilized by NO in normoxic conditions by S-nitrosylation, inhibiting ubiquitination and degradation.^106^

### Carbon monoxide

Before CO was identified as the second gas transmitter, it was known to be produced endogenously as an unimportant byproduct of heme degradation.^107^ It is now clear that the CO generated by heme degradation plays a significant role in cellular signaling.^107^ Heme oxygenase (HO) enzymes are homodimeric proteins that catalyze heme degradation into biliverdin, ferrous iron, and CO.^108^ There are two active isoforms of HO, the inducible expressed HO-1 and the constitutively expressed HO-2.^108^ The third isoform, HO-3, has been identified as inactive.^109^ In the mouse adrenocortical cell line (Y1), HO-1 is induced by adrenocorticotropic hormone in a time and dose-dependent manner, likely through cAMP/PKA signaling pathways.^110^ HO-1 expression is regulated primarily by nuclear factor erythroid 2-related factor 2, AP-1 and HIF.^111^ While the isoforms have many similarities, HO-2 is unique because it is also a hemoprotein containing a heme regulatory motif independent of the heme degradation.^112^ HO-2 is also unique in being induced by glucocorticoids.^113^

Most of CO’s activity in biological systems comes from its ability to bind metals.^97^ Of note is CO’s interactions with the ferrous iron of heme and the regulation of various hemoproteins.^97^ While this theory of CO’s signaling mechanism may at first seem simple, it is further complicated by the hemoprotein hemoglobin, which CO has an extremely high affinity towards (~240-fold greater than that of oxygen) and is the mechanism behind CO poisoning.^114^ Therefore, a majority of endogenous CO is bound to hemoglobin to form carboxyhemoglobin; the average non-smoker has levels of under 2% carboxyhemoglobin.^115^ Binding affinity and stability depend on species; carboxyhemoglobin has a half-life of 20 minutes in rats and mice compared to 5 hours in humans.^116^ While CO has been shown to bind to other hemoproteins, its affinity toward these proteins is significantly lower than towards hemoglobin.^117^ Therefore, the reaction of CO to any select hemoprotein target is likely highly dependent on other hemoproteins within the cellular environment.^117^ Despite this seeming limitation of CO, it has been implicated in regulating several biological mechanisms.^118^,^119^ One prominent example is the CO-mediated increase of cGMP production by binding to the heme group of soluble guanylyl cyclase (sGC), despite its affinity towards sGC being significantly lower than other hemoproteins.^118^ This disconnect between binding affinities and observed biological effects hasn’t been conclusively explained. However, some potential explanations may include that high local concentrations of endogenous CO drive its physiological changes.^118^ Furthermore, CO is the most stable gas transmitter. It is not easily converted into other chemical species and does not rely on the presence or absence of oxidizing or reducing agents to bind metalloproteins.^97^ It is also possible that CO may interact with an unknown allosteric regulator, such as an endogenously occurring analog of the benzyl indazole derivative YC-1.^118^ YC-1 stabilization of sGC’s active configuration enhanced CO-dependent stimulation of sGC, achieving levels similar to NO.^120^

### Hydrogen sulfide

H_2_S was the most recent addition to the gas transmitter family upon the realization that it meets the criteria for inclusion.^93^ H_2_S is endogenously produced primarily by the enzymes cystathionine β-synthase (CBS) and cystathionine γ-lyase (CSE).^121^ These enzymes catalyze the desulfhydration of cysteine, producing H_2_S as part of the cysteine metabolism.^121^ The activity of the sulfurtransferase enzyme 3-mercaptopyruvate sulfurtransferase also produces H_2_S.^122^ Some H_2_S is also produced nonenzymatically by the reduction of sulphur as a byproduct of glycolysis.^123^ The expression of H_2_S-producing enzymes is species and tissue-specific, with levels of CBS, CSE and 3-mercaptopyruvate sulfurtransferase varying across species and tissue types.^121^,^124^ Through negative feedback, elevated H_2_S levels inhibit CSE activity and limit the production of cysteine.^93^ Additionally, exogenous H_2_S donation regulates the transcription of CSE depending on dose, with lower dosages decreasing expression and higher dosages increasing expression, while extremely high doses ultimately inhibit expression.^125^ CBS is allosterically regulated by SAM, increasing its turnover rate.^126^ CBS contains a heme group which acts as a redox sensor, modulating its activity in response to redox potential.^126^

H_2_S primarily acts through the per-sulfhydration of target proteins.^97^ H_2_S differs from NO and CO in that it can be ionized in the physiological pH range, and bisulphide (HS-) is the predominant ion.^97^ Depending on protein interactions, select cysteine thiols can be highly susceptible to sulfhydration by H_2_S.^97^ The sulfhydration of thiols (-SH) into hydropersulfides (-SSH) increases their chemical reactivity, as well as making them more active hydrogen donors in low pH environments.^127^ Sulfhydration affects a large variety of proteins, with proteomic analysis indicating that approximately 10–25% of liver proteins are sulfhydrated under physiological conditions.^128^ For example, sulfhydration of the glycolytic enzyme glyceraldehyde 3-phosphate dehydrogenase, increases enzyme activity.^128^ Similar to NO, H_2_S is often converted into more stable compounds such as thiosulfate and polysulfides,^129^ which can later regenerate into active H_2_S.^101^

## Catecholamines and Gas Transmitters

The molecular crosstalk between catecholamine and gas transmitters has been well established in the literature. The gas transmitters NO, CO, and H_2_S have been implicated in the regulation of catecholamine release both neuronally and hormonally. NO has been implicated as a potent neuromodulator, particularly in regulating dopamine release, with NO being a putative effector molecule in the development of morphine dependence.^130^ NO has been shown to inhibit basal catecholamine efflux from isolated canine adrenal glands.^131^ Chronic blockade of NOS by treatment with N-nitro-L-arginine methyl ester resulted in elevated blood pressure and plasma catecholamine levels in rats at rest and during immobilization stress.^132^ NO has been implicated in the regulation of stimulated release of catecholamines. Treatment of opossums with the NOS inhibitor L-NG-nitro-arginine attenuated the hypogastric nerve-stimulated release of norepinephrine, epinephrine and dopamine from sympathetic nerves innervating the internal anal sphincter; this attenuation was reversed with the addition of L-arginine.^133^ Conversely, in the rat mesenteric arterial bed, periarterial nerve stimulation in the presence of N-nitro-L-arginine methyl ester increased norepinephrine levels, which were reversed with the addition of L-arginine.^134^ It was suggested that NO differentially regulates the stimulated release of catecholamines from sympathetic nerves, likely as a consequence of differences in site, mode of stimulation, and signaling pathways involved. NO can act presynaptically via sGC/cGMP-dependent signaling to induce release; in the periarterial nerve, the mechanism of NO acts independently of sGC.^134^,^135^ In isolated canine adrenal glands, catecholamine release induced by the nicotinic cholinergic agonist 1,1-dimethyl-4-phenylpiperazinium iodine (DMPP) was increased by the non-selective NOS inhibitor N(G)-monomethyl-L-arginine and the selective neuronal NOS inhibitor 7-nitroindazole.^136^ Treatment with N-nitro-L-arginine methyl ester and N-monoethyl-L-arginine also ameliorated the inhibition of DMPP-induced catecholamine release when endothelial and chromaffin cells were co-cultured.^137^ In perfused rat striatum, L-NG-nitro-arginine inhibited endomorphin-1-induced dopamine release.^138^ This suggests that NO plays differential roles in neuronal and endocrine catecholamine release, with its effect likely depending on the specific mechanism triggering catecholamine release. DMPP induces Ca^2+^ influx, driving catecholamine release,^138^,^139^ while endomorphin-1 acts through μ-opioid receptor signaling cascades to drive catecholamine release.^140^

Work investigating the effects of CO and catecholamines began with CO poisoning. Plasma epinephrine and norepinephrine increase significantly in rats exposed to CO poisoning.^141^ A study examining catecholamine content in carotid bodies in response to high acute CO concentrations found increased dopamine levels under normoxic conditions.^142^ In contrast, another study exposed rats to lower, though still high levels of CO and found no effect on carotid dopamine or norepinephrine levels.^143^ CO has been shown to inhibit the uptake of synaptosomal dopamine in a time and dose-dependent manner.^144^ Exposure to pure CO has been shown to increase dopamine release from rat striatum.^145^ CO exposure administered to mimic the amounts typically inhaled during cigarette smoking showed no change in epinephrine or norepinephrine levels compared to air.^146^ The increase in catecholamine release at high levels of CO can be attributed to carboxyhemoglobin formation and resulting tissue hypoxia.^147^,^148^ More promising are recent studies on low-dose CO exposure and modulation of HO. CO has been identified as a key modulator of dopamine neurotransmitter release.^149^ Low-dose exposure to CO enhanced survival and dopaminergic differentiation in human neural stem cells.^150^ This exposure increased the proportion of TH-expressing catecholaminergic neurons.^150^ Inhibition or knockdown of HO in the postsynaptic mushroom body neurons in *Drosophila melanogaster* inhibited CO production and presynaptic dopamine release.^151^ It highlights the importance of CO in neuronal dopamine release. A similar relationship between CO and dopamine may be present in the gut microbiome,^152^ but further research is required to confirm CO-dependent dopamine secretion in specific bacterial species. How endogenous CO modulates norepinephrine and epinephrine release, specifically from adrenal tissue, has not been significantly investigated and should be the focus of future studies.

The effects of H_2_S on catecholamine release have been well described.^153^ In rat adrenal chromaffin cells, H_2_S induces catecholamine release by inhibiting membrane depolarization,^154^ and in calf chromaffin cells, exogenous H_2_S donation increased Ca^2+^ levels, triggering exocytosis.^155^ However, in neonatal rat adrenal chromaffin cells, H_2_S-induced catecholamine release was faster and smaller in quantal size than similarly induced release from adult rat adrenal chromaffin cells.^156^ This suggests that the effect of H_2_S on catecholamine release changes across development. Endogenously produced H_2_S stimulates the secretion of catecholamines from rainbow trout (*Oncorhynchus mykiss*) adrenal chromaffin cells in response to hypoxia.^157^ The effects of exogenous H_2_S on isolated porcine iris-ciliary body catecholamine content and release have been studied.^158^ H_2_S donation by sodium hydrosulfide (NaHS) or sodium sulfide (Na_2_S) inhibited electrically evoked norepinephrine release.^158^ NaHS treatment caused concentration-dependent decreases in norepinephrine and epinephrine but not dopamine.^158^ In a similar study, concentrations of NaHS greater than 100 µM reduced dopamine and norepinephrine levels in the porcine iris–ciliary body.^159^ NaHS also decreased dopamine and norepinephrine levels in the rat cortex.^159^ These studies highlight the differential role of gas transmitters when produced and tightly regulated at endogenous levels, as opposed to the rapid, non-physiological, concentrated exogenous delivery at a physiological level. Consequently, the slow, sustained release of H_2_S in *Caenorhabditis elegans* has been shown to reduce reactive oxygen species (ROS) stress and increase dopamine levels.^160^ Taken together, NO, CO and H_2_S are key modulators of catecholamine release. Given the high degree of crosstalk between these gas transmitters, it is likely that they co-regulate catecholamine release; future studies should aim to describe how these gas transmitters work in tandem to regulate catecholamine release. Future work should also examine the differential regulation of catecholamine release by NO, CO, and H_2_S in neuronal and hormonal contexts.

Compared to the role of gas transmitters on catecholamine release, the role of gas transmitters in regulating catecholamine production is less defined. Exogenous NO donation has been shown to increase the expression of *Th*, *Dbh*, and *Pnmt*.^161^ Additionally, NO potentiated the response of *Th*, *Dbh*, and *Pnmt* to glucocorticoid and cholinergic antagonists.^161^ The specific activators and inhibitors of kinases confirmed the role of PKA, PKC, and protein kinase G in the NO-mediated induction of genes responsible for catecholamine biosynthesis.^161^ Ansell et al.^161^ provided evidence that catecholamine biosynthesis genes are highly responsive to NO signaling. Conversely, exogenous donation of CO has been shown to decrease *Th* and *Dbh* expression while slightly increasing *Pnmt* expression.^162^ This suggests that NO and CO have opposing roles in the regulation of catecholamine biosynthesis gene expression. Exogenous CO donation has also been shown to attenuate hormonal and neuronal induction of catecholamine biosynthesis gene expression.^162^ Interestingly, exogenous H_2_S donation does not affect catecholamine biosynthesis gene expression.^162^ The current work on the effects of gas transmitters on catecholamine biosynthesis genes is limited to exogenous donation with quick-release donors at short time points. Future studies should investigate the long-term effects of NO, CO, and H_2_S on catecholamine biosynthesis using slow-release donors or by modulating endogenous production. Gas transmitters have been shown to play differential roles across systems, depending on release rate and donor concentrations.^163^,^164^,^165^ Catecholamines have also been shown to induce the expression of some endogenous gas transmitter enzymes. Catecholamines enhance LPS-induced mRNA expression of inducible NOS in macrophages, while also increasing L-arginine uptake and NO production.^166^ Epinephrine has been shown to activate endothelial NOS via phosphorylation.^167^ Dopamine has been shown to induce the expression of HO-1.^149^,^168^,^169^ Epinephrine increases HO activity in hepatocytes.^170^ Overall, these studies highlight the complex relationship between catecholamine and gas transmitters signaling pathways. Future work should investigate the molecular mechanism by which these pathways interact.

## Hypoxic Signaling, a Potential Link between Catecholamines and Gas Transmitters

One system implicated in the etiology of hypertension, the regulation of catecholamine and gas transmitter signaling, is hypoxia. Obstructive sleep apnea (OSA) is a disease characterized by episodes of intermittent hypoxia due to partial or complete blockage of the upper airway during sleep and is the most common cause of secondary hypertension.^171^ Roughly half of all OSA patients also present with hypertension.^172^ Hypoxia induced by OSA triggers increased sympathetic nerve activity, systemic inflammation, and metabolic dysregulation.^171^ The increased sympathetic nerve activity is accompanied by increased circulating catecholamines.^173^,^174^ Sympathetic nerve activity remains raised while OSA patients are awake, suggesting an adaptation to repeated hypoxic exposure.^175^ While OSA is the largest cause of secondary hypertension, only a small portion of cases of hypertension can be linked to an underlying disease.^176^ Nevertheless, these results highlighted the importance of understanding the role of hypoxia in the etiology of hypertension, specifically concerning catecholamines.

The effects of hypoxia on adrenal catecholamine production and release have been well documented. The exposure of rats to chronic intermittent hypoxia increases the release of catecholamines from the adrenal medulla.^177^ Treatment with antioxidants ameliorated hypoxia-induced catecholamine release, implicating ROS-mediated signaling.^177^ Additionally, exposure of isolated mouse adrenal medulla slices to chronic intermittent hypoxia increased the readily releasable pool of secretory granules, thereby increasing the secretory capacity of catecholamines.^178^ The increase in the readily releasable pool of secretory granules was driven by ROS-mediated PKC signaling.^178^ Hypoxia has also been shown to induce the expression of catecholamine biosynthesis genes. Exposure of PC12 cells to hypoxic conditions induces expression of *Th* and increases *Th* mRNA stability^179^ and induces expression of *Dbh* and *Pnmt*.^75^ Exposure of PC12 cells to hypoxic conditions also induces the expression of HIF1α and increases nuclear localization and HIF1α-HRE binding complexes.^89^ Both HIF1α and HIF2α affect the *Th* promoter,^51^ in addition to AP-1 and CREB proteins.^40^,^50^ Expression of AADC is also increased in response to hypoxia.^76^ HIF2α is the key regulator of AADC mRNA expression in response to hypoxia, and also plays a key role in the development of catecholaminergic systems.^57^ HIF2α activity drives the cellular response to chronic hypoxic exposure.^180^ The differential regulation between TH and AADC in response to hypoxia serves an important role in maintaining catecholaminergic homeostasis under hypoxic conditions.^57^ Upregulation of AADC by HIF2α in response to chronic hypoxia prevents the accumulation of L-DOPA driven by increased acute hypoxic TH expression.^57^ While no HREs have been identified in the *Dbh* promoter, the knockdown of HIF2α drastically reduces the expression of *Dbh*.^57^ The response of DBH and PNMT to hypoxia is mediated post-transcriptionally by miRNA-375 in Neuro-2a and NB-1 cells.^76^ miRNA-375 binds to DBH and PNMT mRNA, resulting in translational suppression and degradation.^76^ The levels of miRNA-375 are significantly decreased in response to hypoxia.^76^ The response of *Pnmt* to hypoxia in PC12 cells is also driven by HIF1α induction of Egr1 and Sp1 rather than coactivation.^89^ miRNA-375 has also been shown to regulate catecholamine biosynthesis by targeting Sp1 mRNA.^181^ Additionally, ROS has synergistic effects on both hormonal and neuronal signaling of catecholamine biosynthesis gene expression.^182^ Oxidative stress also plays a role in the catecholamine-mediated development of hypertension.^183^ A study has linked prenatal exposure to glucocorticoids to increased expression of *Pnmt* and the development of adult hypertension in rats.^184^ A similar increase in mRNA levels of *Pnmt* transcriptional regulators, Egr1, AP-2, and GR, was also observed.^184^ Interestingly, co-treatment with antioxidants attenuates the prenatal glucocorticoid exposure-mediated increases in catecholamine biosynthetic gene expression and the resultant rise in blood pressure.^183^ This highlights the importance of ROS signaling in the regulation of catecholamine biosynthesis genes and the development of hypertension.

Endothelial cells also play an essential role in regulating local vascular tone. In response to local stimuli, including hypoxia, endothelial cells release vasodilators and vasoconstrictors.^185^ The endothelial response to hypoxia is primarily driven by changes in the levels and DNA-binding activities of several transcription factors, including AP-1, nuclear factor-κB, and HIF1.^185^ Hypoxia in the short term causes endothelial cells to release vasoconstrictors such as endothelin-1, while long-term hypoxia causes vascular remodeling.^185^ The production and release of catecholamines have also been observed in endothelial cells.^186^ *Th*, *Dbh* and *Pnmt* expression are all observed in bovine and mouse endothelial cells, and their expression is increased by exposure to hypoxic conditions.^186^ It highlights another important potential site for the study of HIF1α-mediated catecholamine regulation.

NO, CO, and H_2_S have all been implicated in the regulation of cellular response to hypoxia.^187^ NO has been shown to inhibit hypoxic pulmonary vasoconstriction via a cGMP-dependent mechanism.^188^ HO-2 has been identified as an O_2_ sensor.^189^,^190^ HOs catalyze three oxidation steps to degrade heme, each requiring O_2_; therefore, hypoxic conditions significantly decrease the efficiency of HOs.^191^ While an alternate H_2_O_2_-mediated oxidation of the rate-limiting step has been described,^192^ CO production by HO-2 notably decreases under hypoxic conditions.^193^ As described above, CO inhibits CSE expression.^194^ Under hypoxic conditions, HO-2 produces less CO, thereby reducing its inhibition of CSE expression.^195^ Increased H_2_S activates carotid body signaling.^195^ A similar response is observed in CBS expression, except the increase in H_2_S supports the vasodilation of precapillary arterioles.^196^

One significant component of hypoxia-mediated signaling is the HIFα transcription factors.^197^ In normoxic conditions, HIF1α undergoes hydroxylation by prolyl hydroxylases, resulting in its degradation via the proteasome. In hypoxic conditions, the O_2_ levels are inadequate for hydroxylation, leading to the accumulation of HIF1α.^197^ This accumulation allows HIF1α to dimerize with HIF1β and translocate to the nucleus, where it can bind to HRE in the promoter region of select genes.^197^ Gas transmitters have been shown to differentially interact and regulate HIF1α signaling in both normoxic and hypoxic conditions (**Figure 3**). Under normoxic conditions, the exogenous donation of NO using the spontaneous donors 1-substituted diazen-1-ium-1,2-diolate (NOC-18) and S-nitrosoglutathione stabilizes and increases HIF1α protein expression, leading to increased transcriptional activity.^106^ NO stabilizes HIF1α by S-nitrosylation of the HIF1α protein directly or S-nitrosylation of the Von Hippel–Lindau protein, preventing binding and, therefore, proteasomal degradation.^198^

**Figure 3:**
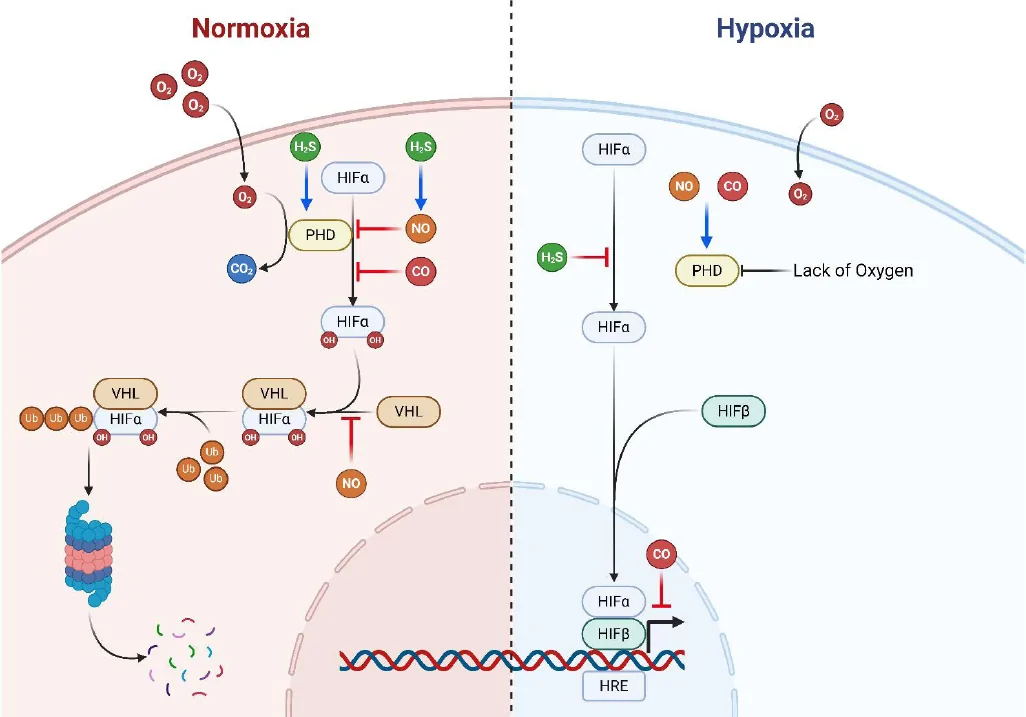
Gas transmitters interact and regulate HIF1α signaling in both normoxic and hypoxic conditions. Under normoxic conditions, HIFα subunits are rapidly degraded by PHD proteins, marking them for destruction by the VHL E3 ubiquitin ligase. Under hypoxic conditions, the low O_2_ availability inhibits PHD activity, allowing HIFα to accumulate and dimerize with HIFβ to bind DNA and activate transcription. Regulation of the HIF oxygen-dependent degradation pathway by gas transmitters depends on the cell’s O_2_ state. Created in BioRender. Dingley R (https://BioRender.com/t70gqdi) is licensed under CC BY 4.0. HIF: Hypoxia-inducible factor; HRE: hypoxia response element; PHD: prolyl hydroxylase domain; Ub: ubiquitin; VHL: Von Hippel-Lindau.

Under hypoxic conditions, NO prevents the stabilization of HIF1α by inhibiting mitochondrial respiration and increasing prolyl hydroxylase-dependent degradation of HIF1α.^199^,^200^ NO has been shown to activate prolyl hydroxylases enzymatic activity in hypoxic conditions.^201^ Similarly, NO prevented hypoxia-mediated HIF1α stabilization in Hep3b cells and human umbilical vein endothelial cells.^202^ Interestingly, only the NO donor sodium nitroprusside affected hypoxia-mediated HIF1α accumulation, with the other two donors, the nitrates nitroglycerin, and isosorbide dinitrate had no effect.^202^ This difference may result from the requirement for enzymatic conversion of nitroglycerin and isosorbide dinitrate, compared to the direct release of NO by sodium nitroprusside.^203^ These fundings suggest that differences in donor release time may determine the effect of NO under hypoxic conditions, it may also highlight the possibility that sodium nitroprusside may have additional effects outside of NO release.

Like NO, CO affects HIF1α differently depending on the available oxygen content. In human umbilical vein endothelial cells, under normoxic conditions, exogenous CO donation using tricarbonyldichlororuthenium(II) dimer, Ru_2_Cl_4_(CO)_6,_ also known as CO releasing molecule 2, increases HIF1α levels by both translational activation and stabilization of HIF1α protein.^204^ The increase in HIF1α protein is mediated by a CO-dependent increase in HIF1α/HSP90 interactions.^204^ These results were also reproduced *in vivo* using a mouse model of ischemia-reperfusion injury, wherein pretreatment with CO stabilized HIF1α and protected against ischemia-reperfusion injury. Taken together, these studies provide two separate mechanisms for the normoxic stabilization of HIF1α by CO.

Under hypoxic conditions, CO has been shown to have the opposite effect on HIF1α. CO has been shown to inhibit the hypoxia-mediated activity of HIF1α by reducing protein accumulation.^205^ Similarly, NO reduced HIF1α protein accumulation.^205^ CO releasing molecule 2 treatment attenuated the hypoxia-mediated upregulation of Hif1α mRNA induced by acute reflux esophagitis in rats,^206^ and when induced by peritoneal adhesion.^207^ Additionally, HIF1α has been shown to induce the expression of HO-1.^208^ Both NO and CO suppress the hypoxic induction of HIF1α-mediated expression of vascular endothelial growth factor, decreasing binding to the HRE in its promoter region.^209^ A cGMP analog mimicked this effect.^209^ In addition, NO-mediated cGMP production also inhibits hypoxia-mediated HIF1α protein accumulation.^210^

Similar to NO and CO, H_2_S affects HIF1α depending on oxygen content. HIF1α is a vital transcription factor in Caenorhabditis elegans response and survival to H_2_S.^211^ In human umbilical vein endothelial cells, H_2_S donation using NaHS increased HIF1α protein levels and translational expression.^212^ The miRNA, miRNA-640, inhibits HIF1α mRNA by binding to the 3′-untranslated regions, and H_2_S downregulates the expression of miRNA-640 through the vascular endothelial growth factor receptor 2-mammalian target of rapamycin pathway.^212^ Stabilization of HIF1α by NaHS has also been observed in a rat model of cardiac arrest and cardiopulmonary resuscitation.^213^ In a mouse model of ischemic vascular remodeling, H_2_S treatment increases endothelial cell HIF1α expression and activity in a NO-dependent manner.^214^ This occurred through increased endothelial NOS expression and liberation of NO from nitrite stores.^214^ This highlights cross-talk between these two gas transmitters.

NaHS has been shown to inhibit the hypoxia-mediated accumulation of HIF1α by increasing the phosphorylation activity of eukaryotic translation initiation factor 2α.^215^ Under hypoxic mimetic conditions, the use of GYY4137, a slow-releasing H_2_S donor in human hepatocellular carcinoma cells, inhibited hypoxia-mediated increases in HIF1α levels.^216^ Interestingly, the enzymatic activity of CBS and the resultant H_2_S produced persulfidates prolyl hydroxylase 2, increasing enzymatic activity under normoxic conditions, supporting hydroxylation and degradation of HIF1α.^217^ Under hypoxic conditions, when H_2_S levels are lower or if CBS activity is inhibited, the persulfidates prolyl hydroxylase 2 remains unpersulfidated, lowering its activity and promoting HIF1α stability.^217^ It is important to note that this mechanism occurs in the opposite direction to all previous effects of gas transmitters and might highlight a difference in H_2_S regulatory activity between endogenous and exogenously donated levels. These studies collectively underscore the dual role of gas transmitters in regulating HIF1α. These gas transmitters appear to modulate the cellular response to oxygen, contrasting with the traditional HIF1α paradigm. They increase HIF1α levels in normoxic conditions while decreasing levels in hypoxic conditions. Additionally, these fundings emphasize the significant crosstalk between these gas transmitters and their comparable modes of action and regulatory effects. Taken together, the investigation of hypoxic signaling, primarily via HIF1α, may be key to elucidating the relationship between hypertension, catecholamines, and gas transmitters.

This review highlights that the cross-talk between gas transmitters and catecholamines is complex and context-dependent. NO, CO, and H_2_S have all been shown to modulate catecholamine release and the gene expression of catecholamine biosynthesis genes through both distinct and overlapping mechanisms, including cGMP-dependent signaling, redox regulation, and hypoxia signaling. These interactions highlight a dynamic regulatory network with significant implications for cardiovascular physiology and the development of hypertension. However, several limitations constrain the current understanding of this field. Much of the evidence reviewed relies on exogenous gas donor compounds, and when endogenous modulation is used, conflicting results are observed. Future research should prioritize the use of models that modulate endogenous production of gas transmitters through genetic or pharmacological means. Additionally, much of the work discussed in the review focuses on the relationship between a single gas transmitter and catecholamines. Given the high degree of cross-talk and overlap between NO, CO, and H_2_S, future studies should investigate the role of these gases as an integrated signaling network. This review covered the interactions between gas transmitters and catecholamine biosynthesis and release; there may also be significant interactions between gas transmitters and catecholamine catabolism, receptor signaling, and downstream intracellular signaling cascades. A deeper mechanistic understanding of these pathways, particularly under hypoxic and disease-relevant conditions, may reveal novel therapeutic targets for the treatment of cardiovascular disorders, including hypertension.

**Facts**

1. Gas transmitters are key regulators of cardiovascular physiology and modulate both neuronal and endocrine catecholamine signaling.
2. The effects of gas transmitters on catecholamine release are tissue-, context-, and concentration-dependent.
3. Hypoxia and hypoxia-inducible factor signaling are a central regulatory axis linking the activity of gas transmitters and catecholamine biosynthesis and release.

**Open Questions**

1. What are the specific molecular mechanisms by which nitric oxide, carbon monoxide and hydrogen sulfide regulate catecholamine biosynthesis and homeostasis under physiological conditions?
2. How do gas transmitters crosstalk to co-regulate catecholamine biosynthesis and release?
3. To what extent is the dysregulation of gas transmitter and catecholamine signaling involved in the development of cardiovascular diseases, including hypertension?
